# *Erratum*: Vol. 65, No. 50-51

**DOI:** 10.15585/mmwr.mm6602a8

**Published:** 2017-01-20

In the report “Characteristics of Electronic Cigarette Use Among Middle and High School Students — United States, 2015,” in the table on page 1426, the percentage and confidence interval for females using only disposable types of e-cigarettes should have been 15.4 (12.6–**18.7**).

